# IL-22 is related to development of human colon cancer by activation of STAT3

**DOI:** 10.1186/1471-2407-13-59

**Published:** 2013-02-05

**Authors:** Runqiu Jiang, Haiyang Wang, Lei Deng, Jiajie Hou, Ruihua Shi, Ming Yao, Yun Gao, Aihua Yao, Xuehao Wang, Lianzhen Yu, Beicheng Sun

**Affiliations:** 1 Liver Transplantation Center, The First Affiliated Hospital of Nanjing Medical University, 300 Guangzhou Road, Nanjing, Jiangsu Province, P.R. China; 2 The key Laboratory of living donor liver transplantation, Ministry of Health, Nanjing, P.R. China; 3 Department of Gastroenterology, The First Affiliated Hospital of Nanjing Medical University, 300 Guangzhou Road, Nanjing, Jiangsu Province, P.R. China

**Keywords:** Colon cancer, Ulcerative colitis, IL-22, TILs, STAT3

## Abstract

**Background:**

It has been previously reported that IL-22, one of the cytokines secreted by Th17 cells, demonstrates both a protective and inflammatory promotion effect in inflammatory bowel disease (IBD) through STAT3 signaling activation. We sought to investigate the role of IL-22 expression in colon cancer (CC).

**Methods:**

The expression of IL-22 and related molecules were detected in human CC, the detail function and mechanism of IL-22 were investigated by in vivo and in vitro model.

**Results:**

Our results demonstrated significant upregulation of IL-22 in human CC tumor infiltrated leukocytes (TILs) compared to peripheral lymphocytes. Moreover, our findings demonstrated that IL-22 expression was significantly higher in ulcerative colitis (UC) tissues versus normal colon tissues. Both IL-22 receptor α1 (IL-22RA1) and IL-23 were highly expressed in CC and UC tissues compared to normal controls. TILs exhibiting various IL-22 expression levels isolated from CC patients were demonstrated to enhance tumor growth and metastasis co-transplanted with Hct-116 cells underwent subcutaneous transplantation in mice model. Tumor growth and metastasis was promoted by STAT3 phosphorylation and upregulation of its downstream genes such as Bcl-xl, CyclinD1, and VEGF. In vitro studies confirmed the anti-apoptotic and pro-proliferation effect of IL-22 according to the BrdU cooperation assay and peroxide induced apoptosis analysis with or without the presence of IL-22.

**Conclusion:**

In this study we demonstrated that excessive IL-22 in the CC and UC microenvironment leads to tumor growth, inhibition of apoptosis, and promotion of metastasis depend on STAT3 activation.

## Background

Colon cancer (CC) is a common and lethal cancer that exhibits regional variation in incidence and mortality around the world. Globally, CC is the third most commonly diagnosed cancer in males and the second in females, with over 1.2 million new cases and 608,700 deaths every year [1]. Ulcerative colitis (UC) is one of the risk factors for CC. In 1988 it was reported that sustained UC over a period of 8–10 years significantly increases the risk of CC by 0.5–1% [2]. Furthermore, after 40 years of UC, approximately 25–30% of patients will have developed CC [3]. Both UC and CC exhibit a strong link with an inflammatory microenvironment composed of a large population of normal and premalignant intestinal epithelial cells (IECs) or tumor cells, immune cells, macrophages etc.; the determinants of the pro- or anti-tumor effect within the microenvironment have become central in cancer research [4].

Moreover, much of the growth that stimulates the cross-talk between immune and IECs or malignant cells is mediated by cytokines that activate the oncogenic transcription factor STAT3 [5], a major intrinsic activator in cancer inflammation and a regulator of the tumor microenvironment [6,7]. STAT3 induces the expression of genes important for cell cycle progression (such as cyclinD1 and PCNA) as well as suppression of apoptosis (Bcl-XL, Bcl-2, and Mcl-1), eventually promoting cell survival and proliferation during colitis-associated tumorigenesis [7-9]. It has been previously demonstrated that specific ablation of STAT3 in intestinal epithelial cells suppresses cell proliferation and reduces tumor incidence in both a DSS-induced colitis model and colitis associated cancer (CAC) model [10,11]. Furthermore, enhanced STAT3 activation has been shown to exhibit an accelerating effect on CAC development by mutation of the gp130 receptor [10].

The most famous STAT3 activator, IL-6, is widely recognized to play an inflammation-related role in colon cancer. In recent years, the involvement of IL-6 in UC and CC has been an area of active investigation in both experimental animal models and clinical research [12-15]. Within the microenvironment of CC or UC, IL-6 originates from various cells including IECs and immunocytes, such as Th17 cells. Th17 cells represent a novel subset of T helper cells that exhibit high expression of RORγT and secrete cytokines, including IL-17A, IL-17 F, IL-21, and IL-22 [9,16-19]. Naïve T cells can be recruited and differentiate into Th17 cells that are polarized by IL-23 in concert with IL-1β, IL-6, IL-23, TNF-α, and TGF-β, which is abundant in both UC and CC [20-23]. The role played by Th17 cells in tumor pathogenesis is controversial and remains unclear. On the one hand, IL-17 has been demonstrated to be a valuable marker associated with poor prognosis that promotes angiogenesis via stimulation of VEGF production of cancer cells in CC [24,25]. Abundant Foxp3+/IL-17+ T cells have been detected in CC tissues that exhibited the capacity to drive cancer initiation in cells with high levels of phosphorylated Akt and MAPK [26]. On the other hand, endogenous IL-17 has been shown to reduce tumor growth and lung metastasis due to the effects of IFNγ + NK and IFNγ + tumor-specific T cells; these results suggest that IL-17 potentially promotes protective tumor immunity [27]. Therefore, further investigation concerning the role of Th17 cells in CC is warranted.

As one of the cytokines secreted by Th17 cells, the majority of CC related cytokine studies have reported that IL-22 is not centrally involved. However, IL-22 was demonstrated to exhibit general intestinal antimicrobial defense, regeneration, and protection against injury in a series of studies by Wolk et al. [28]. Recently, the rs1179251 SNP in IL-22 was demonstrated to be associated with risk of colon cancer [29]; Nagakawa et al. demonstrated that IL-22 does not directly act on immunocompetent cells, and artificially IL-22 expression in CC cell line can favor apothanasia of inoculated hosts [30]; An additional two studies were conducted to investigate the role of IL-22 in UC that revealed that IL-22 played a pivotal role in UC pathophysiology through both DMBT1 and REGα [31,32]. However, the specific role of IL-22 in CC remains unclear. Therefore, we sought to investigate IL-22 expression and the related molecules in human CC in order to further elucidate whether IL-22 acts as a tumor promoter and to investigate the underlying mechanisms of action.

## Methods

### Patients

A total of 82 CC tissues and 40 UC tissues were investigated in this study, these tissues were obtained from patients at the time of surgical resection or endoscopy. Normal colon tissues were obtained from 40 Chinese patients who had suffered from non-tumor diseases such as tediously long Colon and vascular malformation. All of the cases occurred between January 2010 and August 2011 at the First Affiliated Hospital of Nanjing Medical University (Nanjing, China), and all the samplers were conducted according to the principles expressed in the Declaration of Helsinki. Written documented informed consent for gene expression analyses of all tissues was obtained from all patients prior to surgery or endoscopy examination; this study and consent procedure was approved by the local ethics committee of the First Affiliated Hospital of Nanjing Medical University. The diagnosis and staging of colon cancer was assessed according to the AJCC (TNM) Staging System. The detailed clinical characteristics of the 82 colon cancer patients were listed in Table 1.

**Table 1:** Clinical characteristics of the 82 colon cancer patients

**Patient characteristics**	**Patients**	P value*
No. of patients	82	ND
Age, year (median, range)	43, (28–77)	ND
Gender		0.52
Male	40	
Female	42	
Histological grade (WHO)		0.07
G1	20	
G2	41	
G3	21	
pT status		0.21
1	7	
2	21	
3	46	
4	8	
pN status		0.17
0	65	
1	17	
pM status		0.19
pMX	70	
pM1	12	
Clinical stage		0.2
I	23	
II	25	
III	21	
IV	13	

ND: not determined.

* by the student's t-test or the Mann–Whitney *U* test.

### Isolation and culture of human CC infiltrated leukocytes

The fresh tumor tissues samples were collected from 82 cases of histologically confirmed colon cancer obtained from hospitalized cancer patients who underwent surgery. The tumor pieces were washed twice in RPMI 1640 (Invitrogen, CA). The fatty, connective, and/or necrotic tissues were removed from the tumor mass in a 10 cm dish. Next, the tissue was cut into 1–2 mm pieces in RPMI 1640, and the minced tumor pieces were transferred into a 15-ml or a 50-ml conical tube and incubated with a triple enzyme digestion medium that contained DNase (30 U/ml), hyaluronidase (0.1 mg/ml), and collagenase (1 mg/ml) for 2 hours at room temperature with gentle shaking. The samples were then resuspended in 10 ml RPMI 1640, filtered through a 70-μm cell strainer (BD), placed into several wells containing 1 ml of T-cell growth medium (RPMI 1640 with 10% human AB serum, supplemented with 5 U/ml human rIL-2) in a 24-well plate, the TILs was processed immediately for IL-22 detection by flow cytometry. To obtain IL-22(+)TILs, lymphocytes were isolated by flow cytometry, and maintained in T-cell growth medium. A polarization stimulation to Th22 cells was performed according Th22 condition described as previous report [33] which can be briefly described as 50 ng/ml TNF-α, 20 ng/ml IL-6, 5 μg/ml anti–IL-12, and 5 μg/ml anti–IL-4 for 5 days, further mixed with Macrophage and NK cells isolated from TILs. We nominated this cell mixture as IL-22 (+) TILs, and preparing for in vitro and in vivo assays.

### In vivo tumorigenesis assay

All animal experiments were performed in accordance with the guidelines of the Animal Care Committee, Nanjing Medical University. Immunodeficient nude mice (5–6 weeks of age) were purchased from Charles River Laboratories, China. All operation concerning animal was performed according to ARRIVE (Animal Research: Reporting of In Vivo Experiments) guidelines. Three groups, with 5 mice each, were injected subcutaneously with three cell mixtures respectively: Hct-116 cells alone, and Hct-116 cells plus TILs obtained from each of two CC patients (ratio 1000:1), respectively, at cell density of 3 × 10^6^ cells in 300 μl saline solution. Animals were sacrificed 6 weeks after transplantation, and the animals were monitored regularly for tumor occurrence throughout the entire experiment period.

### Quantitative real-time PCR

Reverse transcription reactions were performed using the SuperScript First-Strand Synthesis System (Invitrogen, CA), and the RNA templates were treated with DNase to avoid genomic DNA contamination. To determine the relative level of cDNA in the reverse transcribed samples, real-time PCR analyses were performed using an Applied Biosystems 7300 Detection System (Applied Biosystems, CA). The primers sequence were Forward Primer: GCTTGACAAGTCCAACTTCCA, Reverse Primer: GCTCACTCATACTGACTCCGTG, with 140 bp amplification length for human IL-22, and Forward Primer: AAGGTGAAGGTCGGAGTCAAC, Reverse Primer: GGGGTCATTGATGGCAACAATA, with 102 bp amplification length for human GAPDH. The primers were synthesized by Genscript Inc. (Nanjing, China). Real-time PCR reactions were performed in accordance with the instructions of the SYBR® Premix Ex Taq™ kit. (Takara, Japan). Data were normalized with the GAPDH levels in the samples.

### Western blot

Proteins were extracted from cell and mouse tissues and quantified using a protein assay (Bio-Rad Laboratories, CA). Protein samples (30 μg) were fractionated by SDS-PAGE and transferred to a nitrocellulose membrane. Immunoblotting was conducted using antibodies against IL-22, IL-22RA1, total STAT3, p-STAT3(S727), Bcl-xl, CyclinD1, and VEGF (all purchased from Abcam Inc, MA). The results were visualized via a chemiluminescent detection system (Pierce ECL Substrate Western blot detection system, Thermo Scientific, IL) and exposure to autoradiography film (Kodak XAR film).

### Immunohistochemistry (IHC)

All the tissues were removed and fixed in 4% paraformaldehyde overnight at 4°C, processed, and sectioned at 5 μm thickness. The sectioned slides were stained immunohistochemically for IL-22, IL-22RA1, IL-23, CEA, and p-Stat3 (S727) (all purchased from Abcam Inc., MA) using techniques described previously [34].

### Flow cytometry

The peripheral lymphocytes and TILs were stimulated at 37°C for 5 hours with Leukocyte Activation Cocktail (BD Pharmingen). Thereafter, cells were stained with surface markers, fixed and permeabilized with IntraPre Reagent (Beckman Coulter), and finally stained with intracellular markers. Data were acquired on FACSVantage SE and analyzed with CellQuest software. The fluorochrome-conjugated mAbs against CD3, CD4, CD8, CD14, CD16, and CD56 are purchased from BD Pharmingen (CA).

### BrdU cooperation and immunofluorescense assay

Hct-116, Hct-116 + TILs, Hct-116 + IL-22(+)TILs, Hct-116 + IL-22(+)TILs + IL-22mAb, Hct-116 + IL-22(+)TILs + IL-6mAb (antibodies was all purchased from Abcam Inc., MA. 10 μg/ml anti-IL-22 or anti-IL-6 3 hours prior to the assay) and Hct-116 + IL-22(+)TILs + WP1066 (EMD Chemical USA, MA, 5uM for the concentration of WP1066) were cultured on coverslips such that they were rapidly dividing. Cells were incubated with 100 μM BrdU for 2 hours, the medium aspirated, and immediately fixed and permeabilized in cold methanol:acetone (1:1), then blocked with PBS/3% BSA (Sigma-Aldrich, MO), and incubated with primary BrdU monoclonal antibody (Sigma-Aldrich) diluted in 3% BSA/PBST (0.2% Triton X-100). Following incubation with rabbit anti-mouse FITC-conjugated secondary antibody (Sigma-Aldrich), slides were mounted in Mounting Medium (with 1.5 μg/ml DAPI) (Santa Cruz), and visualized with a fluorescense microscope (Axiovert 200; Zeiss, Stuttgart, Germany).

### Peroxide induced apoptosis and flow cytometry analysis

Hct-116, Hct-116 + TILs, Hct-116 + IL-22(+)TILs, Hct-116 + IL-22(+)TILs + IL-22mAb, Hct-116 + IL-22(+)TILs + IL-6mAb and Hct-116 + IL-22(+)TILs + WP1066 (same treatment to BrdU cooperation assay) were cultured with complete medium with 0.5 mM peroxide overnight. Next, the cells were harvested and fixed with 70% cold EtOH at −20°c overnight, then further analyzed by flow cytometry (FACSCaliburTM; BD Biosciences, NJ) using a PI/Annexin staining kit (Invitrogen, CA).

### Statistical analysis

The results are expressed as mean ± SD. Comparisons between two groups were performed using the student’s *t*-test or the Mann–Whitney *U* test, as appropriate. All statistical analyses were performed using SPSS statistical software (version 13.0), and two-tailed t-tests were applied to all data unless otherwise specified, with P < 0.05 considered to represent a statistically significant result.

## Results

### Excessive IL-22 in tissues of colon cancer and ulcerative colitis

**Figure 1:**
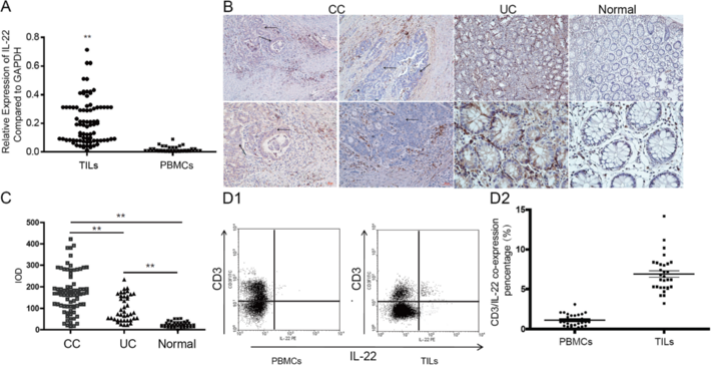
**Elevated expression of IL-22 was investigated in human colon cancer (CC). A**: Expression of IL-22 in PBMCs (n = 40) and TILs (n = 82) from patients suffering from CC, as detected by real-time PCR (unpaired *t*-test). **B**: Average integrated optical density (IOD) was obtained by analyzing five fields for each slide evaluated by Image-Pro Plus software (version 5.0) for IHC staining of IL-22 in CC, UC and normal controls (unpaired *t*-test). **C**: Expression and distribution of IL-22 in normal colon tissues (n = 40), CC tumor tissues (indicated by arrows) (n = 82) and UC tissues (n = 40), as analyzed by IHC. **D1**: IL-22 expression in TILs (n = 32) and PBMCs (n = 32) by flow cytometry co-stained with antibody to CD3, **D2**. Comparison of IL-22 in TILs and corresponding PBMCs (paired *t*-test). ** represented P < 0.01 and * represented P < 0.05.

In order to investigate the expression of IL-22 in human colon cancer, tumor infiltrated leukocytes (TILs), which are the principal source of IL-22, were isolated from excised fresh tumor tissues. The total RNA was extracted from the TILs followed by the cDNA synthesis. Significant high expression of IL-22 were detected by real-time PCR in the TILs samples compared to peripheral blood mono-nuclear cells (PBMCs) (P = 0.00618, P < 0.01, by unpaired *t*-test) (Figure 1A). However, no significant difference was found between IL-22 expression and varied groups of clinical characteristics for colon cancer patients (Table 1). The distribution and localization of IL-22 proteins was verified by IHC in 82 cases of CC tissues, 40 cases of normal colon tissues, and 40 cases of UC tissues in which there was potential chronic inflammation for CC. IL-22 existed in the peri-tumor or non-parenchymal tissues around the intestinal gland area in most CC tissues (71 positive in a total of 82 cases, 71/82) and UC tissues (31/40), whereas normal colon tissue samples were almost all negative (5/40) (Figure 1B), which indicated that IL-22 acted as a driver of inflammation based on previous reports in IBD patients [35]. Similar to the findings of real-time PCR analysis, all slides evaluated by software “Image-plus pro” (Ver. 5.0) also exhibited a significantly higher positive rate in tissues of both CC and UC compared to normal colon tissues (CC vs. Normal P = 0.00021, P < 0.01, UC vs. Normal P = 0.0062, P < 0.01, and CC vs. UC P = 0.0075, P < 0.01, by unpaired *t*-test) (Figure 1C). Furthermore, high expression of IL-22 in CC patients was also confirmed by flow cytometry (TILs vs. PBMC P = 0.00068, by paired *t*-test) (Figure 1D1, D2).

**Figure 2:**
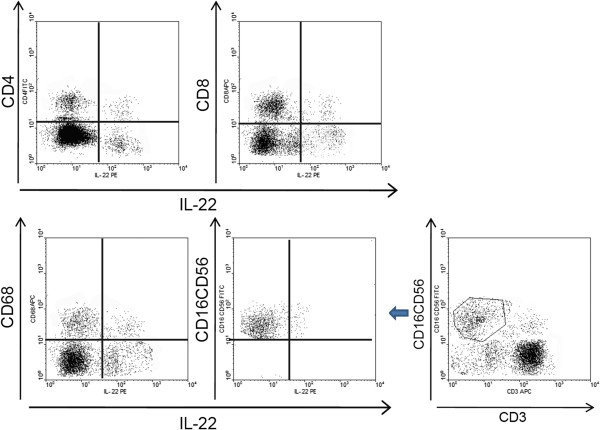
A representative case of IL-22 expression in TILs (n = 31) by flow cytometry co-stained with antibody to CD4, CD8, CD68, and CD3 + CD16 + CD56.

However, TILs are white blood cells originated from the blood stream, they are composed of various of inflammatory cells, including lymphocytes, macrophages, natural killer cells (NK) etc. Therefore, in order to identify the phenotypic features of cells expressing IL-22 in colon cancer, IL-22 was co-stained with other cellular surface markers such as CD3, CD4, CD8, CD68, and CD3 + CD16 + CD56 in 32 cases of TILs isolated from human colon cancer tissues. This finding indicated that both CD4 and CD8 positive lymphocytes expressed IL-22 (4.80 ± 1.81% for CD4 positive cells and 4.01 ± 1.72% for CD8 positive cells), and a small portion of NK cells were positive for IL-22 (3.01 ± 1.29%%). A small population of macrophages (CD68 positive cells, 3.92 ± 1.12%) unexpectedly secreted IL-22 (Figure 2). We sought to isolate IL-22 expressing TILs for further functional studies in accordance with the distribution of IL-22 in TILs. Lymphocytes, macrophages, and NK cells were isolated by flow cytometry respectively. Lymphocytes were maintained in vitro after polarizing stimulation to Th22 [33], and then further mixed with macrophages and NK cells into a cell mixture, we nominated this cell mixture as IL-22(+) TILs.

### IL-22 related proteins are also over-expressed in human colon cancer and ulcerative colitis

**Figure 3:**
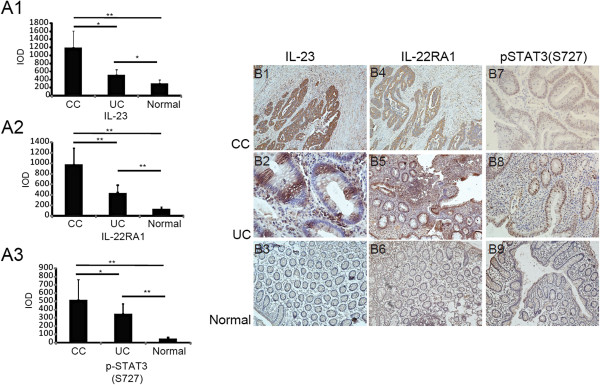
**IL-22 related proteins are also over-expressed in human colon cancer and ulcerative colitis. A1-A3**: Average integrated optical density (IOD) was obtained by analyzing five fields for each slide evaluated by Image-Pro Plus software (version 5.0) for IHC staining of IL-23, IL-22RA1, and p-STAT3 (S727) in human CC, UC, and normal colon tissues. **B1-3, B4-6** and **B7-B9**: Expression and distribution of IL-23, IL-22RA1 and p-STAT3 (S727) in human CC (n = 84), UC (n = 40) and normal colon tissues (n = 40) respectively (Mann–Whitney *U* test). * represented P < 0.05 and ** represented P < 0.01.

Because IL-22 is one of the hallmark cytokines secreted by Th17 cells, we analyzed three essential cytokines in the microenvironment that recruit and induce Th17 proliferation. Numerous studies have verified the level of IL-1β and TNF-α in human colon cancer [20-23]. Our findings indicated that expression of IL-23, which has been rarely reported previously, was detected by IHC analysis in both CC and UC. These results indicated that significant upregulation of IL-23 was detected in both CC and UC (CC vs. Normal control, P = 0.0027, P < 0.01, and UC vs. Normal control, P = 0.011, P < 0.05, CC vs. UC, P = 0.017, P < 0.05, by Mann–Whitney U test), whereas relatively weak expression was detected in only a small portion of normal colon tissue samples (4/40) (Figure 3A1, B1-B3). The positive region of IL-23 in CC and UC was confined mostly to tumor and intestinal epithelial cells. In addition to IL-23, we identified another key molecule, IL-22RA1, which is necessary for signal transmission; the localization was similar to IL-23 and it was identically overexpressed in tumor and intestinal epithelial cells of UC (CC vs. Normal control, P = 0.0044, P < 0.01, and UC vs. Normal control, P = 0.0084, P < 0.01, CC vs. UC, P = 0.0072, P < 0.01, by Mann–Whitney U test), whereas it was nearly negative in normal colon tissues (7/40)(Figure A2, B4-B6). As the downstream effects of IL-22, activation of STAT3 was accessed by staining with phosphorylated STAT3 at the residue of S727. STAT3 was activated in both UC (34/40) and CC (68/82), which is a significantly higher rate compared to its activation in normal colon tissue (3/40) (CC vs. Normal control, P = 0.0051, P < 0.01, and UC vs. Normal control, P = 0.0081, P < 0.01, CC vs. UC, P = 0.022, P < 0.05, by Mann–Whitney U test) (Figure 3A3, B7-B9).

### IL-22 is related to promoted tumor growth and metastasis in vivo

**Figure 4:**
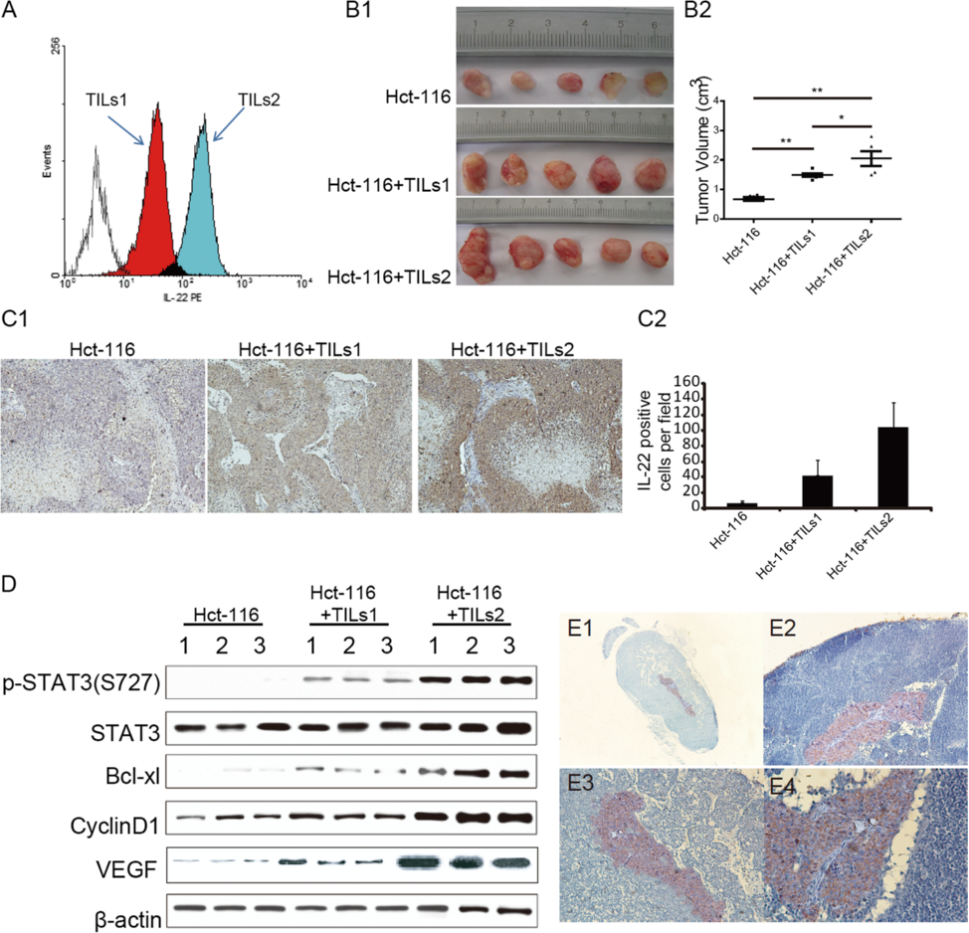
**IL-22 promotes tumor growth and metastasis in vivo.** TILs were isolated from two CC patients and co-transplanted subcutaneously into nude mice with Hct-116 cells. Tumor growth and metastasis was investigated. **A**: Different IL-22 expression levels of TILs1 and TILs2 analyzed by FCMs. **B1**: Tumor tissues obtained from nude mice from each group. **B2**: Comparison of tumor volume of each group (unpaired t-test). **C1**: IL-22 positive cell distribution in tumor tissues of each group investigated by IHC. **C2**: Average integrated optical density (IOD) was obtained by analyzing five fields for each slide evaluated by Image-Pro Plus software (version 5.0) for IHC staining of IL-22. **D**: Western-blot detection of expression of p-STAT3 (S727), total STAT3, Bcl-xl, CyclinD1, and VEGF, all normalized to β-actin for every 3 samples in each group. **E**: IHC staining of CEA in lymph nodes around the tumor beneath the skin of nude mice. E1 (×40), E2, E3 (×100) and E4 (×200), ** represented P < 0.01.

To investigate the effect of IL-22 on tumor growth and metastasis, we designed an in vivo tumorigenesis assay using the subcutaneous cell transplantation model. Hct-116, a colon cancer cell line, was co-transplanted with TILs isolated from two CC patients with different IL-22 expression levels according to analysis by FCMs (Figure 4A). Cell suspensions consisting of a 1:1000 cell mixture ratio (TILs: Hct-116) were injected subcutaneously into BALB/c nude mice. TILs from two CC patients promoted tumor growth and metastasis. Significant increase in tumor volume was evident when Hct-116 cells were co-transplanted with TILs compared to Hct-116 cells alone; moreover, our findings indicated that elevated IL-22 expression was correlated to enhanced tumor volume (TILs1, 1.63 ± 0.23 cm^3^ vs. Hct-116, 0.34 ± 0.19 cm^3^, P = 0.0019, P < 0.01; TILs2, 2.01 ± 0.30 cm^3^ vs. Hct-116, P = 0.0048, P < 0.01, TILs1 vs. TILs2, P = 0.022, P < 0.05 by unpaired *t*-test; Figure 4B1 and B2). Tumor tissue was analyzed by IHC with IL-22 staining, it exhibited that IL-22 expressing TILs isolated from two patients have proliferated underneath the mice skin (Figure 4C1, C2). Increased phosphorylation of STAT3 (S727) in tumor tissues was detected; upregulation of cyclinD1 was also found to be the effect of the activation of STAT3 signaling, which potentially explains the tumor growth enhanced by TILs. BCL-XL, an anti-apoptosis gene, was similarly elevated in the tumor tissues with co-transplanted with TILs, which were also downstream transcripts of STAT3 activation and potentially explains the tumor growth enhancement (Figure 4D).

We investigated metastasis by analyzing the lymph nodes that emerged around the tumor tissue (2/6 in the TILs1 group and 4/6 in the TILs2). We confirmed that Hct-116 invaded the lymph nodes with IHC staining for carcino-embryonic antigen (CEA), which is negative in normal lymph nodes (Figure 4E). No visceral metastasis was found in all groups. Based on this result, we analyzed VEGF expression in the tumor tissues, and our findings indicated that VEGF expression was elevated in correlation with TILs (Figure 4D); increased VEGF expression is also a downstream target of STAT3 activation.

### IL-22 enhances tumor proliferation and anti-apoptotic ability by activating STAT3 signaling in vitro

**Figure 5:**
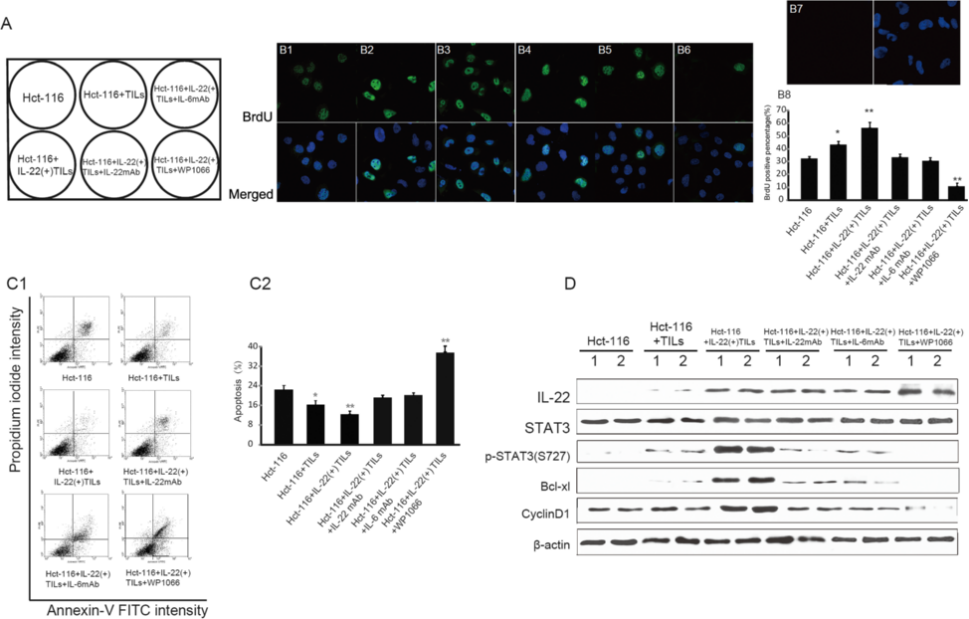
**IL-22 enhances tumor proliferation and anti-apoptosis ability by activating STAT3 signaling in vitro. A**: Schematic diagram of experimental design. **B1-B6**: Immunofluorescence staining of BrdU in green and DAPI in blue, reflecting cell proliferation in each group detected by fluorescence microscopy (×200). **B7**: Immunofluorescense staining with non-specific antibody control (NC) (×200). **B8**: Comparison of cell proliferation in each group (the independent co-cultures experiments were repeated in triplicate) (unpaired *t*-test). **C1**: Analysis of apoptosis of Hct-116 cells induced by peroxide in each group by flow cytometry, **C2**: Comparison of apoptosis in each group (the independent co-cultures experiments were repeated in triplicate) (unpaired *t*-test). **D**. Western-blot detection of IL-22, p-STAT3 (S727), total STAT3, Bcl-XL, Bcl-2, and CyclinD1 expression, all normalized to the β-actin. *p <0.05; **p < 0.01.

To further investigate the mechanism of IL-22 on tumor growth, we utilized in vitro studies (Figure 5A). Hct-116 cells were co-cultured with TILs and IL-22(+)TILs. In order to exclude the effect of IL-22 secreted by IL-22(+)TILs, IL-22 was blocked, moreover, the effect of TILs secreted IL-6 which can induce activation of STAT3 was blocked as well, in addition, the expression of both IL-22 and IL-6 from TILs and IL-22(+)TILs were verified by ELISA in the supernatant of their cell culture (Additional file 1: Figure S1). The effects of IL-22 on tumor cell proliferation and anti-apoptosis were then investigated. The proliferation capability of each group was assessed by the BrdU cooperation assay. Both TILs and IL-22(+)TILs significantly enhanced the proliferation ability of Hct-116 cells (Hct-116 + TILs vs. Hct-116, P = 0.026, P < 0.05; Hct-116+ IL-22(+)TILs vs. Hct-116, P = 0.0072, P < 0.01, by unpaired t-test) (Figure 5B1-B3, B8); however, when IL-22 and IL-6 were blocked, the percentage of proliferating cells (BrdU positive cells) decreased dramatically (Figure 5B4, B5 and B8). Additionally, CyclinD1 expression also decreased in correlation with fewer dividing cells (Figure 5D). IL-22(+)TILs and TILs also significantly enhanced the anti-apoptosis ability of Hct-116 cells, which decreased the percentage of apoptotic cells significantly induced by peroxide in comparison to Hct-116 cells (Hct-116 + TILs vs. Hct-116, P = 0.032, P < 0.05; Hct-116+ IL-22(+)TILs vs. Hct-116, P = 0.0086, P < 0.01, by unpaired t-test) . However, when the effect of IL-22 and IL-6 were blocked by their antibody, apoptosis increased (Figure 5C). To explain this, the anti-apoptosis gene BCL-XL increased with the existence of TILs and IL-22(+)TILs, and decreased when IL-22 was blocked (Figure 5D). To further confirm the effect of IL-22 is depend on STAT3 activation, WP1066 which is a STAT3 specific inhibitor was used, decreased proliferation and increased apoptosis were investigated even with the existence of IL-22(+)TILs (WP1106 vs. Hct-116 for BrdU cooperation assay P = 0.0031, P < 0.01, and for apoptosis assay, P = 0.0047, P < 0.01, by unpaired t-test) (Figure 5B6, B8 and C). Evidently, all of these changes were originated from the activation or blocking of STAT3 signaling. Therefore, in this study, we introduced a STAT3 activator, IL-22, that exhibits a tumor enhancement mechanism similar to IL-6.

## Discussion

It has been previously demonstrated that IL-22 plays a protective role within many organs, such as anti-microbial defense, regeneration, and protection against injury [36,37]. These findings are based on the distribution of two reporters: IL-22R1 (IL-22RA1) and IL-10R2. IL-22R1 is located primarily in the skin, digestive tract (including pancreas and liver), lung, and kidney, while IL-10R2 is ubiquitously expressed [28,36]. IL-22 acts as a protector in intestinal mucosal healing through multiple signaling activation steps. In an intestinal infection with *C. rodentium*, IL-22 is produced earlier than IL-17A and it plays a decisive role, whereas IL-17A does not [38]. Moreover, Sugimoto et al. demonstrated that IL-22 contributes to rapid amelioration of local inflammation associated with Th2-mediated colitis [39]. Additionally, studies in genetically engineered mice have demonstrated that epithelial STAT3 activation in dextran sodium sulfate colitis is dependent upon IL-22 rather than IL-6, and that both IL-22 and epithelial STAT3 is important in wound-healing experiments in vivo [40]. Therefore, according to these studies, most of the protective roles of IL-22 in the intestinal tract are linked to STAT3 in IECs, a pleiotropic transcription factor with important functionality in cytokine signaling in a variety of tissues [41,42]. However, IL-22 has also been considered an inflammatory driver in IBD based upon both clinical evidence and mouse model data. Highly elevated serum levels and a potential systemic role for IL-22 have been demonstrated to correlate with disease severity in patients with Crohn’s disease (CD). In 2005 it was shown that IL-22 was much higher in UC compared to CD, and a study of human IBD revealed that IL-22 derived from activated T cells acts on human colonic subepithelial myofibroblasts to stimulate secretion of proinflammatory cytokines and matrix-degrading molecules, thus demonstrating its proinflammatory/remodeling role in IBD [35]. Similar outcomes have been obtained from a colitis mouse model, indicating that highly elevated IL-22 expression was an inflammation driver in either a direct or indirect manner [39,43]. More recently, the role of IL-22 in mouse IBD and colon cancer have been clarified by Huber et al. which indicated that the ratio of IL-22/IL-22BP is critical in regulating intestinal tissue repair and tumorigenesis in the colon [44].

As discussed above, STAT3 activation by IL-22 plays both protective and inflammatory driver roles in human IBD, which is similar to what occurs in the liver. IL-22 demonstrates a directly protective role in acute liver injury, which has been demonstrated by both the Radaeva and Zenewicz research groups. However, the authors also hypothesized that the protective effect of IL-22 was due to activation of STAT3, and that its anti-apoptotic and regeneration promoting effect potentially contributes to the development of HCCs [45,46]. This hypothesis was verified by both us and Park et al. recently, and it has been revealed that IL-22 exhibits opposing short-term and long-term effects in the liver which in turn promote cell proliferation, survival, metastasis, and transformation from chronic hepatitis to HCC [34,47]. Therefore, we investigated whether the opposing effects of short-term and long-term IL-22 activated STAT3 exist in the human intestinal tract. Initially, our investigation confirmed that massive levels of IL-22 were present in the UC tissues of the Chinese population. Secondly, we reported for the first time that excessive IL-22 also was present in human colon cancer. Furthermore, up-regulation of IL-22 in TILs derived from human CC was associated with the activation of STAT3 and exhibited tumor promotion and enhancement of metastasis in both in vitro and in vivo model.

The tumor microenvironment is composed of tumor cells, macrophages, and immunocytes etc. where the interactions between these cells involves their secreted cytokines and consists of a free-forward loop with persistent activation of STAT3 enabling promotion of tumor growth [7]. Consistent with IL-23 and IL-22RA1, an IL-22 feed-forward loop in the CC or UC microenvironment has been demonstrated through our research. The IL-22 signal can be transmitted through a heterodimeric receptor complex that consists of IL-22R1 (IL-22RA1) and IL-10R2 [48,49]. Unlike the ubiquitously expressed IL-10R2 chain, the IL-22RA1 chain was normally restricted to non-immune cells such as epithelial cells and hepatocytes [50]. In correlation with overexpression of IL-22 in TILs of CC, IL-22RA1 is also overexpressed in colon cancer cells and in IECs of UC, which ensures the transmission of the IL-22 signal. Pro-tumor cytokine IL-23, which is also regulated by STAT3 [51,52], is over expressed in CC and UC. Excessive expression of IL-23 plus TNF-α, IL-6, and IL-1β, which have already been demonstrated to be overexpressed in human CC, composed a milieu for infiltrated naive lymphocytes which were also enrolled by STAT3 activation to differentiate to Th17 cells expressing IL-22 [20-23]. In this study, we transplanted this IL-22 related tumor microenvironment beneath the skin of nude mice. The proliferation and metastasis enhancing effects of this free-forward loop were confirmed, and proliferation associated cyclinD1, cell survival associated BCL-XL, and metastasis associated VEGF were all upregulated and mediated by STAT3 activation, which was demonstrated by phosphorylation of S727 residue [9,52]. Furthermore, these tumor growth and metastasis promotional effects of IL-22 were demonstrated to occur in a dose-dependent manner when various tumor microenvironments were transplanted with a range of IL-22 levels. We investigated more precise and accurate mechanisms with in vitro studies utilizing the IL-22 and IL-6 depletion assay; cell survival and tumor promotion was eliminated when IL-22 was blocked based upon the attenuated activation of STAT3 which is similar to IL-6, Moreover, the specialty of IL-22 effect through STAT3 signaling was verified by using STAT3 inhibitor WP1066.

## Conclusion

This study revealed a previously unknown role of IL-22 in human UC and CC, which potentially enhances proliferation, cell survival, and metastasis. Further studies are warranted to investigate the potential of anti-IL-22 therapeutics for the prevention and treatment of human UC and CC.

## Competing interests

The authors declare that they have no competing interests.

## Authors’ contributions

Conceived and designed the experiments: BS, RJ, LY and XW. Performed the experiments: RJ, HW, LD, JH and MY. Analyzed the data: RJ, HW, LD, JH, MY, RS, YG, and AY. Wrote the paper: RJ. All authors read and approved the final manuscript.

## Pre-publication history

The pre-publication history for this paper can be accessed here:

http://www.biomedcentral.com/1471-2407/13/59/prepub

## Supplementary Material

Additional file 1: Figure S1 Secretion of IL-22 and IL-6 in TILs and IL-22+ TILs isolated from human colon cancer. TILs and IL-22+ TILs were isolated or induced by the method described in “Methods” obtained from 3 colon cancer patients. IL-22 and IL-6 secretion in the supernatant of TILs and IL-22+ TILs were detected by commercialized ELISA Kits. Click here for file
