# Supplementary material for: IL-22 is related to development of human colon cancer by activation of STAT3
Source: BMC Cancer. 2013 Feb 5;13:59. doi: 10.1186/1471-2407-13-59 (PMC3607898; doi:10.1186/1471-2407-13-59)
Supplement: Additional file 1: Figure S1 — Secretion of IL-22 and IL-6 in TILs and IL-22+ TILs isolated from human colon cancer. TILs and IL-22+ TILs were isolated or induced by the method described in “Methods” obtained from 3 colon cancer patients. IL-22 and IL-6 secretion in the supernatant of TILs and IL-22+ TILs were detected by commercialized ELISA Kits. [file 1471-2407-13-59-S1.doc]

Supplementary Figure 1


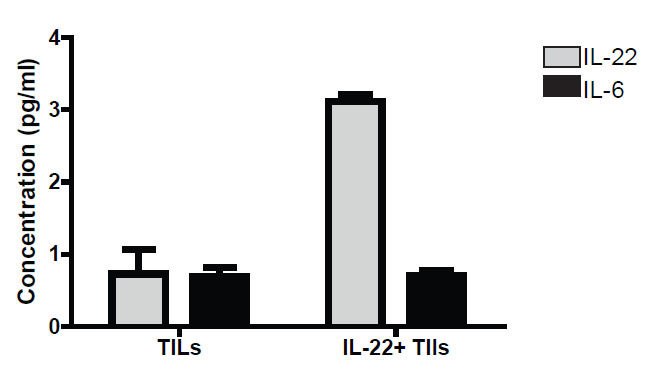


**Figure legend**:

Secretion of IL-22 and IL-6 in TILs and IL-22+ TILs isolated from human colon cancer.

TILs and IL-22+ TILs were isolated or induced by the method described in “Materials and Methods” obtained from 3 colon cancer patients. IL-22 and IL-6 secretion in the supernatant of TILs and IL-22+ TILs were detected by commercialized ELISA Kits.
